# Global Trends in the Use of Insecticides to Control Vector-Borne Diseases

**DOI:** 10.1289/ehp.1104340

**Published:** 2012-01-17

**Authors:** Henk van den Berg, Morteza Zaim, Rajpal Singh Yadav, Agnes Soares, Birkinesh Ameneshewa, Abraham Mnzava, Jeffrey Hii, Aditya Prasad Dash, Mikhail Ejov

**Affiliations:** 1 Laboratory of Entomology, Wageningen University, Wageningen, the Netherlands; 2 Vector Ecology and Management, Department of Control of Neglected Tropical Diseases, World Health Organization, Geneva, Switzerland; 3 World Health Organization, Regional Office for the Americas, Washington, DC, USA; 4 World Health Organization, Regional Office for Africa, Harare, Zimbabwe; 5 World Health Organization, Regional Office for the Eastern Mediterranean, Cairo, Egypt; 6 World Health Organization, Regional Office for the Western Pacific, Manila, Philippines; 7 World Health Organization, Regional Office for South-East Asia, New Delhi, India; 8 World Health Organization, Regional Office for Europe, Copenhagen, Denmark

**Keywords:** Chagas disease, dengue, global trend, insecticide resistance, insecticides, insecticide use, integrated pest management, integrated vector management, leishmaniasis, malaria, pesticide management, resistance management, vector control

## Abstract

Background: Data on insecticide use for vector control are essential for guiding pesticide management systems on judicious and appropriate use, resistance management, and reduction of risks to human health and the environment.

Objective: We studied the global use and trends of insecticide use for control of vector-borne diseases for the period 2000 through 2009.

Methods: A survey was distributed to countries with vector control programs to request national data on vector control insecticide use, excluding the use of long-lasting insecticidal nets (LNs). Data were received from 125 countries, representing 97% of the human populations of 143 targeted countries.

Results: The main disease targeted with insecticides was malaria, followed by dengue, leishmaniasis, and Chagas disease. The use of vector control insecticides was dominated by organochlorines [i.e., DDT (dichlorodiphenyltrichloroethane)] in terms of quantity applied (71% of total) and by pyrethroids in terms of the surface or area covered (81% of total). Global use of DDT for vector control, most of which was in India alone, was fairly constant during 2000 through 2009. In Africa, pyrethroid use increased in countries that also achieved high coverage for LNs, and DDT increased sharply until 2008 but dropped in 2009.

Conclusions: The global use of DDT has not changed substantially since the Stockholm Convention went into effect. The dominance of pyrethroid use has major implications because of the spread of insecticide resistance with the potential to reduce the efficacy of LNs. Managing insecticide resistance should be coordinated between disease-specific programs and sectors of public health and agriculture within the context of an integrated vector management approach.

Vector control constitutes an important element in the current global strategies for the control of major vector-borne diseases, notably malaria, dengue, leishmaniasis, and Chagas disease (Townson et al. 2005). Since the advent of DDT (dichlorodiphenyltrichloroethane) and other organochlorine insecticides in the 1940s, vector control has depended largely on the action of chemical insecticides to kill vectors or prevent transmission of disease pathogens to humans. In recent years, vector control interventions in which insecticides are used to control malaria have been scaled up in many countries, resulting in considerable reductions in disease morbidity and mortality [World Health Organization (WHO) 2010a].

Against these positive outcomes, a recent global survey among countries at risk for vector-borne diseases drew attention to critical deficiencies in the capacity to manage vector control insecticides. Deficiencies included the lack of guidelines for pesticide registration, gaps in pesticide procurement practices, and a lack of training of vector control decision makers (Matthews et al. 2011; van den Berg et al. 2011). These shortcomings could hamper the optimal selection and use of insecticides and application methods for vector control, undermining the effectiveness and safety of operations.

The extensive use of vector control insecticides has raised concern over the development of insecticide resistance and adverse effects on the environment and human health. Genes conferring insecticide resistance have been spreading in vector populations, particularly in vectors of pathogens causing malaria and dengue (Ranson et al. 2010, 2011). Recent monitoring data on malaria vectors in Africa confirm that levels of resistance are increasing, especially against pyrethroid insecticides, and that resistance is being recorded in new locations (Butler 2011). Pyrethroids are currently the only class of insecticides approved for treating netting fabric because of their rapid effects on mosquitoes at low dosages combined with their relatively low health risk (Zaim et al. 2000). Factory-made long-lasting insecticidal nets (LNs) currently are a key malaria control tool; thus, the effectiveness of pyrethroids must be preserved for as long as possible (WHO 2011a).

The Stockholm Convention on Persistent Organic Pollutants (2012) emphasized the need for alternatives to the use of the organochlorine compound DDT in vector control, given its toxicity, environmental persistence, bioaccumulation, and potential for transboundary movements. Until locally appropriate and cost-effective alternatives are available for a sustainable transition from DDT, WHO (2007) recommends using it for indoor residual spraying, according to their guidelines and recommendations and those of the Stockholm Convention (2012), and using best practices to protect spray workers and residents in treated households from exposure (WHO 2011b).

The goals of this study were to provide a comprehensive assessment of global use patterns of vector control insecticides, to determine whether trends in the use of pyrethroids (for applications other than LNs) are consistent with the need to preserve the effectiveness of LNs in areas where they are widely deployed, and to determine if the global use of organochlorines for vector control has changed since the Stockholm Convention went into effect in 2004. We limited the scope of our analysis to insecticides used against vectors of pathogens that cause human disease. In addition, we included insecticides used to (re)treat nets or curtains but excluded insecticides used in the manufacture of LNs.

## Materials and Methods

*Data collection.* We asked countries to provide national data on insecticide use for vector control using a standard reporting format (WHO 2011c) to obtain data on insecticide compound and class, formulation and concentration, type of application, disease targeted or purpose of use, and the amount of formulation used during each year. We converted quantitative data on insecticide formulations to the amount of the active ingredient in each formulation for comparative purposes.

In October 2010, we sent the survey to the WHO regional offices for Africa, the Americas (South and North America), the Eastern Mediterranean, Europe, South-East Asia, and the Western Pacific.

These offices in turn distributed the survey to WHO country offices in member states, specifically their focal points for malaria and other vector-borne diseases, that worked through ministries of health to facilitate data collection and validation.

Member states targeted for this study were those with vector control programs in place. Territories or special areas that are the responsibility of other member states were excluded from the survey. In total, 143 countries were selected, representing a human population of 5.49 billion. The survey excluded Australia, Japan, Canada, and the United States, and targeted countries in the European region were limited to Armenia, Azerbaijan, Georgia, Kyrgyzstan, Tajikistan, Turkey, Turkmenistan, and Uzbekistan. However, because some of the excluded countries use insecticides for vector control, for example, against West Nile virus in North America (Kramer et al. 2008), these countries should be considered for inclusion in future studies.

Countries were asked for insecticide use data for 2008 and 2009 and to provide missing or updated data, where appropriate, for the years 2000 through 2007. The data were supplemented with data previously reported to the WHO using the same format and reporting system.

*Insecticide application methods.* We classified the reported methods of application of vector control insecticides as residual spraying, space spraying, treatment of nets, and larviciding (WHO 2006). Residual spraying refers to the spraying of interior surfaces of houses targeting indoor resting vectors (or indoor residual spraying), and “perifocal treatment” of larval habitats and peripheral mosquito resting surfaces for dengue control (WHO 2009a). Treatment of nets refers to the conventional application of insecticides to treat bed nets or curtains (excluding the use of insecticides in factory manufacturing of LNs). Larviciding refers to the use of insecticides to treat aquatic breeding sites of mosquitoes.

Miscellaneous application methods reported by some countries but excluded from the analysis were dusting treatment against plague (1.4% of total use) and the use of insecticidal paints (0.1% of total pesticide use).

We quantified the insecticide application rate for residual spraying as the amount of active ingredient per square meter. We distinguished between two categories of insecticides: organochlorines, organophosphates, and carbamates, for which the recommended application rate for residual spraying is generally around 1.5 g/m^2^ (although there are exceptions, for example, the carbamate bendiocarb, with a recommended rate of 0.1–0.4 g/m^2^) (Najera and Zaim 2002), and pyrethroids, which have a recommended application rate of around 0.025 g/m^2^, 1/60th of the amount of the first category. A similar conversion factor applies to insecticides commonly used in space spraying, which are quantified as the amount of active ingredient applied per hectare (Najera and Zaim 2002). Hence, the “spray utility,” defined here as the recommended surface or area covered by a given amount of active ingredient, was approximately 60 times higher for pyrethroids than for organochlorines, organophosphates, and carbamates.

*Data analysis.* We conducted analyses of the 10-year average data and of annual trends. We assumed that a report by a country for a particular year covered all insecticide uses for vector control during that year. If a country failed to report for a particular year, we treated the data as missing for that country and year.

For each country, we determined the 10-year average use by averaging the use for all reported years, excluding years with missing data. Data from all responding countries were totaled to produce a global estimate.

The rate of reporting increased gradually, from 78 countries in 2000 to 92 countries in 2009. Therefore, we weighted the data to estimate the global annual use. For each year, we determined the combined human population of the responding countries using WHO health statistics (WHO 2010b) and divided the resulting value by the total population of all 143 targeted countries to derive a weighting factor based on the proportion of the targeted population with available data. Subsequently, we divided the reported annual insecticide use by the weighting factor to estimate the global annual insecticide use for vector control. The weighting factor ranged from 0.81 to 0.91.

We calculated the intensity of insecticide use, expressed in the amount of active ingredient used per capita per year, as a measure of per-capita expenditure or potential human exposure within each WHO region.

Data on the four main disease targets for vector control insecticides—malaria, dengue, leishmaniasis, and Chagas disease—were submitted to separate analysis. Some countries reported that insecticide application methods were targeted at more than one disease. This mixed-purpose use of insecticides, which constituted 2% of the global use of organophosphates and 3% of global use of pyrethroids, was included in the assessment of insecticide use per disease.

## Results

*Country responses.* Of the 143 countries targeted, insecticide use data were provided by 125 countries (87%), representing 97% of the total population of all targeted countries (Table 1). Countries varied in their consistency of annual reporting, but 43 countries representing a human population of 3.88 billion submitted all 10 annual reports. A complete list of responding countries and details regarding annual reporting are provided in Supplemental Material, Table 1 (http://dx.doi.org/10.1289/ehp.1104340).

**Table 1:** Countries and their representative populations that were targeted and responded to the data reporting request according to WHO region.*^a^*

No. of countries								
Responded*b*							2008 population (×10^6^)*c*		
WHO region	Targeted*d*	1–4 reports	5–9 reports	10 reports	Total *n* (%)	Targeted	No. responded (%)
Africa		46		19		12		6		37 (80)		805		680 (85)
Americas		32		11		13		8		32 (100)		570		570 (100)
Eastern Mediterranean		21		3		6		9		18 (86)		580		562 (97)
Europe		8		1		0		7		8 (100)		135		135 (100)
South-East Asia		11		1		3		6		10 (91)		1,760		1,737 (99)
Western Pacific		25		7		6		7		20 (80)		1,639		1,629 (99)
All		143		42		40		43		125 (87)		5,489		5,314 (97)
**a**Details on the populations of, and reporting by, individual countries are available in Supplemental Material, Table 1 (http://dx.doi.org/10.1289/ehp.1104340). **b**Countries that responded with 1–4, 5–9, or 10 annual reports over the 10-year period, 2000–2009. **c**Data from WHO (2010b). **d**Canada and the United States (Americas region) and Australia and Japan (Western Pacific region) were not targeted, whereas in the European region, only Armenia, Azerbaijan, Georgia, Kyrgyzstan, Tajikistan, Turkey, Turkmenistan, and Uzbekistan were targeted.														

*Global use.* Malaria was the primary disease target for vector control insecticides, followed by dengue, leishmaniasis, and Chagas disease (endemic to Latin America). Organochlorines and carbamates were used for residual spraying; organophosphates were used for residual spraying, space spraying, and larviciding; and pyrethroids were used for residual spraying, space spraying, and treatment of nets. Space spraying was common only in the American and Western Pacific WHO regions. Very similar amounts of insecticides were used for space spraying against malaria and dengue, which is of concern because space spraying has rather limited indications for malaria control (Najera and Zaim 2002). Additional information on the major insecticide compounds used in the control of malaria and dengue is provided in Supplemental Material, Table 2 (http://dx.doi.org/10.1289/ehp.1104340).

**Table 2:** Average reported insecticide use for vector control according to method of application and class of insecticide by WHO region (2000–2009), in metric tons of active ingredient per year.

Residual spraying							Space spraying			Treatment of nets (PY)*b*	Larviciding*c*		
WHO region*a*	OC	OP	C	PY	OP	PY	OP	PY		
Africa		805		19		19		24		0		0		12		1		0
Americas		0		97		4		164		276		66		0		82		0
Eastern Mediterranean		0		26		5		15		2		5		1		20		1
Europe		0		2		0		1		0		1		0		1		0
South-East Asia		3,623		483		2		39		15		1		4		49		0
Western Pacific		0		1		0		39		292		27		14		9		0
All		4,429		627		30		282		584		100		31		163		2
Abbreviations: C, carbamates; OC, organochlorines (DDT only); OP, organophosphates; PY, pyrethroids. **a**Canada and the United States (Americas region) and Australia and Japan (Western Pacific region) were not targeted, whereas in the European region, only Armenia, Azerbaijan, Georgia, Kyrgyzstan, Tajikistan, Turkey, Turkmenistan, and Uzbekistan were targeted. **b**Conventional application of insecticides to treat bed nets or curtains (excluding insecticides used in factory-made LNs). **c**The use of insecticides to treat aquatic breeding sites of mosquitoes.																		

DDT, the only organochlorine reported, was used in higher quantities than any other insecticide class and was exclusively applied in indoor residual spraying (Table 2). Of the global use of DDT, 82% was in India alone; the remainder was used in Africa, with Ethiopia contributing 11.3%, Mozambique 2.2%, Namibia 1.3%, South Africa 1.2%, and Zimbabwe, Zambia, Madagascar, Eritrea, Swaziland, Uganda, and Mauritius each < 1% of the global use. Organophosphates were used in residual spraying, space spraying, and larviciding. The use of carbamates, which was predominantly in the African region, was small compared with other classes of insecticides. Pyrethroids did not constitute a major global share in terms of metric tons applied but accounted for 81% of the global spray utility (the surface area covered by an active ingredient). Sixty-eight percent of pyrethroids was used for residual spraying, and 24% for space spraying. The American region accounted for 56% of the global amount of pyrethroids used for vector control.

Organochlorines were used at an intensity of 1.18 and 2.09 g per capita per year in the African region and South-East Asian region, respectively. Organophosphate use was most intensive in the American region, at 0.80 g per capita, followed by the South-East Asian region, at 0.33 g per capita. Pyrethroid use was most intensive by far in the American region, at 0.40 g per capita, followed by the African region and Western Pacific region, each at 0.05 g per capita.

In addition to the main classes of insecticides, some countries reported the use of bacterial larvicides (WHO 2011c). *Bacillus thuringiensis israelensis* was reported mainly for dengue control, with a global use of 70–300 metric tons of formulated product per year. *Bacillus sphaericus* was reported exclusively against malaria at an increasing trend with 200–300 metric tons of formulated product in recent years. The reported global use of insect growth regulators was around 3 metric tons of active ingredient per year.

*Individual diseases.* Eighty-one percent of organochlorines (i.e., DDT) was used against malaria (Table 3); the remainder was used for leishmaniasis control in India. Organophosphates and pyrethroids were used mainly against malaria and dengue, and carbamates were used mainly against malaria. Diseases reportedly targeted with pyrethroids were, foremost, malaria and dengue, followed by Chagas disease, leishmaniasis, gastrointestinal diseases, and lymphatic filariasis.

**Table 3:** Average reported insecticide use for vector control according to method of application and class of insecticide by disease (2000–2009), in metric tons of active ingredient per year.

Residual spraying							Space spraying			Treatment of nets (PY)*a*	Larviciding*b*		
Disease	OC	OP	C	PY	OP	PY	OP	PY		
Malaria		3,604		589		26		130		286		11		31		60		0
Malaria and dengue		0		3		0		5		11		33		0		2		0
Malaria and other*c*		0		11		0		4		0		0		0		0		0
Dengue		0		7		1		61		235		34		0		81		0
Dengue and other*d*		0		0		0		0		51		21		0		3		0
Leishmaniasis		825		1		1		20		0		0		0		0		0
Chagas disease		0		5		1		35		0		0		0		0		0
Lymphatic filariasis		0		3		0		1		0		0		0		13		0
Gastrointestinal disease		0		7		0		3		1		0		0		1		0
Bartonellosis		0		2		0		0		0		0		0		0		0
Unspecified		0		1		0		22		0		1		0		2		1
Abbreviations: C, carbamates; OC, organochlorines (DDT only); OP, organophosphates; PY, pyrethroids. **a**Conventional application of insecticides to treat bed nets or curtains (excluding insecticides used in factory-made LNs). **b**The use of insecticides to treat aquatic breeding sites of mosquitoes. **c**Malaria plus leishmaniasis or Chagas disease. **d**Dengue and other arboviral diseases.																		

**Figure 1:**
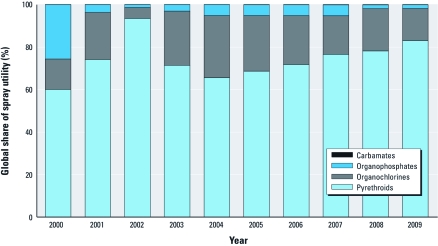
Relative global share of the four classes of insecticides in the annual use for vector control, expressed in terms of spray utility (recommended surface or area covered by a given amount of active ingredient).

*Trends.* The annual data show that global use of the organochlorine DDT has been high, without a clear trend [Table 4; for use by region and disease, respectively, see Supplemental Material, Tables 3 and 4 (http://dx.doi.org/10.1289/ehp.1104340)]. Organophosphate use showed a major decline in 2001 after extensive use during the 1990s, especially in India (WHO 2009b). Carbamate use in indoor residual spraying increased in recent years. Pyrethroid use peaked sharply in 2002 and increased gradually in recent years because of increased use in residual spraying and space spraying. The 2002 peak in pyrethroids was attributable to residual spraying against malaria, dengue, Chagas disease, and leishmaniasis in Brazil that year. The contribution of pyrethroids to global insecticide use for vector control in terms of spray utility increased from 60% in year 2000 to 83% in 2009 (Figure 1).

**Table 4:** Average reported insecticide use for vector control according to method of application and class of insecticide by year (2000–2009), in metric tons of active ingredient.

Application/insecticide class	2000	2001	2002	2003	2004	2005	2006	2007	2008	2009
Residual spraying																				
OC		3,512		5,305		4,814		5,693		5,745		6,065		4,436		4,558		6,170		5,127
OP		5,126		293		135		65		42		115		362		684		145		230
C		12		5		7		8		11		19		18		33		49		30
PY		124		200		1,374		184		153		137		166		176		155		261
Space spraying																				
OP		840		398		934		502		755		875		405		388		284		238
PY		62		71		70		53		54		83		47		111		210		193
Treatment of nets (PY)		58		23		18		29		7		43		11		29		38		14
Larviciding																				
OP		297		180		219		108		188		169		206		221		106		118
PY		1		1		0		0		0		1		6		6		0		0
All																				
OC		3,512		5,305		4,814		5,693		5,745		6,065		4,436		4,558		6,170		5,127
OP		6,263		871		1,288		675		985		1,159		973		1,293		536		585
C		12		5		7		8		11		19		18		33		49		32
PY		245		295		1,462		266		214		264		230		321		403		468
Abbreviations: C, carbamates; OC, organochlorines (DDT only); OP, organophosphates; PY, pyrethroids.																				

**Figure 2:**
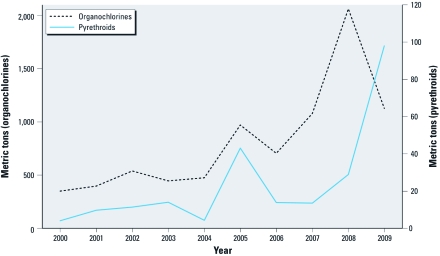
Trends in the annual use of organochlorines and pyrethroids for vector control in the African region (metric tons of active ingredients).

Trends in vector control insecticide use were most marked in the African region. The use of organochlorines (i.e., DDT) increased steadily, peaked in 2008, and dropped in 2009 (Figure 2). The 2009 decline in DDT use was accompanied by an increase in the use of pyrethroids. In India, use of DDT showed a decline from 5,300 to 4,000 metric tons of active ingredient per year in 2004 through 2009. Insecticide use against Chagas disease in the American region was substantially reduced after 2003 [Supplemental Material, Table 4 (http://dx.doi.org/10.1289/ehp.1104340)].

## Discussion

The reported data had exceptional global coverage of human populations living in countries with vector control programs. Nevertheless, some responding countries may have lacked capacity for monitoring and reporting on all uses of vector control insecticides. In particular, dengue control is generally less resourced and structured than is malaria control (Gubler 2002). Hence, inadequate access to data by the national level could have resulted in underreporting of insecticide use for dengue in some countries. Countries with recent programs on West Nile virus control should be considered for inclusion in future studies.

A striking finding was the dominance of pyrethroids for use in vector control, in terms of the area treated, particularly given probable underreporting of use for dengue. The contribution of pyrethroids to insecticide use for vector control increased steadily from 2004. Most apparent was the sharp increase in pyrethroid use in 2009 in the African region, which was predominantly due to indoor residual spraying against malaria. However, it is important to note that our estimates do not account for pyrethroids used in the manufacture of LNs. In 2009, 88 million LNs were distributed in Africa and 13 million were distributed outside Africa; in 2010, 145 million LNs were distributed in Africa and 20 million outside Africa (Milliner J, personal communication, 2011). Assuming equal global shares in the number of LNs manufactured using each of three pyrethroids, and assuming average doses of 1 g/m^2^ for permethrin, 0.07 g/m^2^ for deltamethrin, and 0.23 g/m^2^ for α-cypermethrin and 16 m^2^ of fabric per net (WHO Pesticide Evaluation Scheme, unpublished data, 2011), we estimate that 700 and 1,100 metric tons of pyrethroids were used to manufacture LNs globally in 2009 and 2010, respectively. Consequently, the inclusion of LNs would have more than doubled our 2009 estimate for pyrethroid use in vector control globally.

Concurrent use of pyrethroids for indoor residual spraying and LNs could increase the pressure for resistance development in vector populations (WHO 2011a). In 2009, 19 countries in the African region reported using pyrethroids for indoor residual spraying against malaria. These countries included Ethiopia, Kenya, Liberia, Madagascar, Mali, Mozambique, Nigeria, Rwanda, Senegal, Tanzania, and Uganda, all of which have high coverage rates of LNs for malaria control (WHO 2010a). Hence, the increasing trend in pyrethroid use for indoor residual spraying may not be consistent with the need to preserve the effectiveness of LNs (WHO 2011a).

According to a recent report from the U.S. President’s Malaria Initiative (PMI 2011a), in 2010 and 2011 Ethiopia, Liberia, Mali, Senegal, and Uganda switched partly or entirely from pyrethroids to carbamates (i.e., bendiocarb) for indoor residual spraying because of evidence of resistance against pyrethroids. This move may help preserve susceptibility of vectors to the pyrethroids used in LNs. However, evidence of resistance to bendiocarb has emerged from several countries in Africa (Hunt et al. 2010, 2011; Ranson et al. 2009; Vezenegho et al. 2009).

Pyrethroids also were used against several diseases outside of the African region, including malaria, dengue, leishmaniasis, and Chagas disease in the American region and malaria and dengue in the Western Pacific region. In addition, LNs were distributed in both regions (WHO 2010a). Thus, there is a need to harmonize and coordinate insecticide resistance management between disease-specific programs in all regions, particularly given that the intensity of use of pyrethroids, in terms of grams per capita, was almost eight times greater in the American region than in any other region.

Experience from agriculture and public health indicates that resistance management strategies are most effective when implemented at the onset of control programs to prevent the selection of resistance, rather than responding after resistance genes have already spread [Insecticide Resistance Action Committee (IRAC) 2011]. Australian cotton production provides a good example where effective resistance management necessitated the restriction of pyrethroid use on all crops to a fixed period of 42 days per year, a voluntary strategy that was adopted by almost 100% of growers (Andow et al. 2008; Forrester 1990). This example underscores the rigorous effort that may be required to prevent the spread of resistance genes.

In areas where resistance genes have already spread, immediate implementation of resistance management is required to preserve the effectiveness of available tools (WHO 2011a), notably LNs. Options include using insecticides with different modes of action in rotation, as mixed formulations, or in mosaic patterns (IRAC 2011), but these options are limited by the few distinct modes of action offered by currently available vector control insecticides (Nauen 2007; Zaim and Guillet 2002), by the availability of only one class of insecticides for the manufacture of LNs, and by the fact that LNs can remain efficacious for several years. More cost-effective formulations of existing insecticide molecules are being developed and could be marketable soon, but insecticides with novel modes of action will take much longer to develop (Butler 2011; Hemingway et al. 2006). The use of bacterial larvicides against malaria and dengue signals a diversification of vector control methods in some countries.

Insecticide use in other sectors, particularly agriculture, also contributes to resistance development in disease vectors (Lines 1988). Global insecticide use in 2007 has been estimated at 404,000 metric tons of active ingredient (Grube et al. 2011), with vector control insecticides constituting < 2% of this total. Hence, it would be prudent to coordinate an insecticide resistance management strategy between different sectors using insecticides.

Two patterns in the use of DDT for vector control are apparent since the Stockholm Convention entered into force in 2004. India continued to dominate the global use of DDT but showed a modest decline in use after 2005. In the African region, DDT use increased sharply until 2008, along with efforts to expand programs on indoor residual spraying. In 2009, use of DDT in the African region decreased, primarily due to changes in three countries: Ethiopia reduced the use of DDT after evidence of widespread insecticide resistance (PMI 2011b), Mozambique used up remaining stocks after a change in policy away from DDT (Abilio et al. 2011), and in Uganda a high court decision prohibited the use of DDT in 2008, although the case was later dismissed. The coming years will show whether the recent drop in use of DDT in Africa is part of a trend. New evidence points to resistance to DDT and pyrethroids in parts of Zambia, a country that has been using DDT for malaria control (Chanda et al. 2011).

A long-term strategy to reduce the selection pressure for insecticide resistance and reliance on persistent organic pollutants such as DDT is offered by integrated vector management, including increased collaboration among disease control programs, intersectoral coordination, improved evidence-based targeting, use of multiple vector control methods (including nonchemical methods) wherever practicable, judicious application of insecticides, and implementation of good pesticide management practices (WHO 2012). Integrated vector management was modeled on the positive experience with integrated pest management in agriculture.

## Conclusions

The use of vector control insecticides was dominated by organochlorines (i.e., DDT) in terms of quantity applied (71% of total), and by pyrethroids in terms of surface area treated (81% of total).

The global use of DDT has not changed substantially since the Stockholm Convention entered into force. India, by far the largest user of DDT, showed a modest decline in use after 2005, but use increased sharply in the African region until 2008 because of expanding programs on indoor residual spraying. The increase in pyrethroid use has major implications for the spread of insecticide resistance with potential to reduce the efficacy of LNs, a tool that depends solely on the action of pyrethroids. In the African, American, and Western Pacific regions, pyrethroids were used against several diseases, and in the American region, the use intensity per capita was much higher than in other regions. Insecticide resistance management strategies should be coordinated among disease-specific programs and donor-supported projects and with the agriculture sector. Integrated vector management, as a rational decision-making process, offers a long-term approach to reduce selection pressure for insecticide resistance and to ensure the judicious use of insecticides.

## Supplemental Material

(176 KB) PDF
