# Supplementary material for: Global Trends in the Use of Insecticides to Control Vector-Borne Diseases
Source: Environ Health Perspect. 2012 Jan 17;120(4):577–82. doi: 10.1289/ehp.1104340 (PMC3339467; doi:10.1289/ehp.1104340)
Supplement: (176 KB) PDF [file ehp.1104340.s001.pdf]

Supplemental Material

Global trends in the use of insecticides for vector-borne disease control

Henk van den Berg[1], Morteza Zaim[2], Rajpal Singh Yadav[2], Agnes Soares[3], Birkinesh Ameneshewa[4], Abraham Mnzava[5], Jeffrey Hii[6], Aditya Prasad Dash[7], Mikhail Ejov[8]

[1] Laboratory of Entomology, Wageningen University, Wageningen, the Netherlands

[2] Vector Ecology and Management, Department of Control of Neglected Tropical Diseases, World Health Organization, Geneva, Switzerland

[3] World Health Organization, Regional Office for the Americas, Washington DC, USA

[4] World Health Organization, Regional Office for Africa, Harare, Zimbabwe

[5] World Health Organization, Regional Office for the Eastern Mediterranean, Cairo, Egypt

[6] World Health Organization, Regional Office for the Western Pacific, Manila, Philippines

[7] World Health Organization, Regional Office for South-East Asia, New Delhi, India

[8] World Health Organization, Regional Office for Europe, Copenhagen, Denmark

Address correspondence to:

Dr R.S. Yadav, Vector Ecology and Management, Department of Control of Neglected Tropical Diseases, World Health Organization, 20 Avenue Appia, 1211 Geneva 27, Switzerland. Telephone: 41-22-7912961. Fax: 41-22-7913111. Email: yadavraj@who.int

Table 1. Incidence of reporting by individual countries. Population size is indicated.

WHO Region	Country[a]	Population[b]	Calendar years									
			00	01	02	03	04	05	06	07	08	09
African	Algeria	34.4							1	1	1	1
	Angola	18.0						1	1			1
	Benin	8.7			1	1	1	1			1	1
	Botswana	1.9	1	1	1	1	1	1	1	1	1	1
	Burundi	8.1							1			
	Cameroon	19.1				1	1	1	1	1	1	1
	Cape Verde	0.5	1									
	Congo	3.6	1	1							1	1
	Eritrea	4.9	1	1	1		1	1	1	1	1	1
	Equatorial Guinea	0.7							1			
	Ethiopia	80.7	1	1	1	1	1	1	1	1	1	1
	Gambia	1.7	1			1	1	1				
	Ghana	23.4							1			1
	Guinea	9.8		1	1	1	1	1	1			
	Kenya	38.8		1	1	1	1	1	1	1	1	1
	Lesotho	2.0					1	1	1	1	1	1
	Liberia	3.8										1
	Madagascar	19.1	1	1	1	1	1	1	1	1	1	1
	Malawi	14.8							1	1	1	1
	Mali	12.7	1	1		1	1	1				1
	Mauritania	3.2	1	1	1							
	Mauritius	1.3	1	1	1	1	1	1	1	1	1	1
	Mozambique	22.4						1	1	1	1	1
	Namibia	2.1		1	1	1	1	1	1	1	1	1
	Niger	14.7	1					1			1	1
	Nigeria	151.2							1	1		1
	Rwanda	9.7	1	1	1	1	1	1	1	1	1	1
	Sao Tome & Principe	0.2	1						1			
	Senegal	12.2								1	1	1
	Seychelles	0.1				1	1	1				
	South Africa	49.7			1	1	1	1	1	1	1	1
	Swaziland	1.2						1	1	1	1	1
	Tanzania, United Rep.	42.5							1	1	1	1
	Togo	6.5	1	1	1	1		1				
	Uganda	31.7							1	1	1	1
	Zambia	12.6					1	1	1			
	Zimbabwe	12.5	1	1	1	1	1	1	1	1	1	1
American	Argentina	39.9		1	1	1	1	1	1	1	1	
	Bahamas	0.3		1	1	1						
	Barbados	0.3	1	1							1	1
	Belize	0.3		1	1	1			1	1	1	1
	Bolivia	9.7	1	1	1	1					1	1
	Brazil	192.0	1	1	1	1	1	1	1	1	1	1

Table 1 (cont.)

WHO Region	Country[a]	Population[b]	Calendar years									
			00	01	02	03	04	05	06	07	08	09
American (cont.)	Chile	16.8				1	1	1			1	1
	Colombia	45.0	1	1	1	1	1	1			1	1
	Costa Rica	4.5	1	1							1	1
	Cuba	11.2	1	1	1	1	1	1	1	1	1	1
	Dominica	0.1		1	1	1	1	1		1	1	1
	Dominican Republic	10.0	1	1	1	1	1	1			1	1
	Ecuador	13.5	1	1	1	1	1	1	1	1	1	1
	El Salvador	6.1	1	1	1	1	1	1	1	1	1	1
	Grenada	0.1	1									
	Guatemala	13.7	1	1	1	1	1	1	1	1	1	1
	Guyana	0.8		1	1	1	1	1	1	1	1	1
	Haiti	9.9	1	1	1			1			1	1
	Honduras	7.3	1	1							1	1
	Jamaica	2.7		1	1	1	1	1			1	1
	Mexico	108.6	1	1	1	1	1	1	1	1	1	1
	Nicaragua	5.7	1								1	1
	Panama	3.4	1	1		1	1	1			1	1
	Paraguay	6.2	1	1		1	1	1			1	1
	Peru	28.8	1	1				1				
	Saint Kitts and Nevis	0.1	1									
	Saint Lucia	0.2		1	1	1						
	Saint Vincent & Gren.	0.1	1	1	1	1						
	Suriname	0.5									1	1
	Trinidad and Tobago	1.3	1	1	1	1	1	1	1	1	1	1
	Uruguay	3.3	1	1	1	1	1	1	1	1	1	1
	Venezuela	28.1	1	1	1	1	1	1				
Eastern Mediterranean	Afghanistan	27.2	1	1	1				1	1	1	1
	Bahrain	0.8	1	1	1	1	1	1	1	1	1	1
	Djibouti	0.8	1	1	1					1	1	
	Egypt	81.5							1	1	1	
	Iran, Islamic Republic	73.3	1	1	1	1	1	1	1	1	1	1
	Iraq	30.1					1	1			1	
	Jordan	6.1	1	1	1	1	1	1	1	1	1	1
	Kuwait	2.9	1	1	1	1	1	1	1	1	1	1
	Lebanon	4.2							1	1		
	Morocco	31.6	1	1	1	1	1	1	1	1	1	1
	Oman	2.8	1	1	1	1	1	1	1	1	1	1
	Pakistan	177.0	1	1	1	1	1	1	1	1	1	1
	Saudi Arabia	25.2	1	1	1		1	1	1	1	1	1
	Somalia	8.9		1	1				1	1	1	1
	Sudan	41.3	1	1	1	1	1	1	1	1	1	1
	Syrian Arab Republic	21.2	1	1	1	1	1	1	1	1	1	
	United Arab Emirates	4.5	1	1	1						1	1
	Yemen	22.9	1	1	1	1	1	1	1	1	1	1

Table 1 (cont.)

WHO Region	Country[a]	Population[b]	Calendar years 00	01	02	03	04	05	06	07	08	09
European	Armenia	3.1	1	1	1	1	1	1	1	1	1	1
	Azerbaijan	8.7	1	1	1	1	1	1	1	1	1	1
	Georgia	4.3	1	1	1	1	1	1	1	1	1	1
	Kyrgyzstan	5.4	1	1	1	1	1	1	1	1	1	1
	Tajikistan	6.8	1	1	1	1	1	1	1	1	1	1
	Turkey	73.9	1	1	1	1	1	1	1	1	1	1
	Turkmenistan	5.0	1	1	1							
	Uzbekistan	27.2	1	1	1	1	1	1	1	1	1	1
South-East Asian	Bangladesh	160.0	1						1	1		
	Bhutan	0.7	1	1	1	1	1	1	1	1		
	India	1181.4	1	1	1	1	1	1	1	1	1	1
	Indonesia	227.3	1	1	1	1	1	1	1			
	Maldives	0.3	1	1	1	1	1	1	1	1	1	1
	Myanmar	49.6	1	1	1	1	1	1	1	1	1	1
	Nepal	28.8	1	1	1	1	1	1	1	1	1	1
	Sri Lanka	20.1	1	1	1	1	1	1	1	1	1	1
	Timor-Léste	1.1						1	1	1	1	1
	Thailand	67.4	1	1	1	1	1	1	1	1	1	1
Western Pacific	Brunei Darussalam	0.4				1	1	1			1	1
	Cambodia	14.6	1	1	1	1	1	1	1	1	1	1
	China	1344.9	1	1	1	1	1	1	1	1	1	1
	Cook Islands	0.0		1	1							
	Fiji	0.8	1	1	1	1	1	1	1	1	1	1
	Kiribati	0.1	1			1	1	1			1	1
	Lao PDR	6.2	1	1	1	1	1	1	1	1	1	1
	Malaysia	27.0	1	1	1	1	1	1	1	1	1	1
	Micronesia	0.1							1	1		
	Nauru	0.0							1	1	1	1
	New Zeeland	4.2			1	1	1	1			1	1
	Palau	0.0				1	1	1	1	1		
	Philippines	90.3	1	1	1	1	1	1	1	1	1	1
	Korea, Rep. of	48.2							1	1		
	Singapore	4.6	1	1	1			1	1	1	1	1
	Solomon Islands	0.5		1	1	1	1	1	1	1	1	1
	Tonga	0.1				1	1	1				
	Tuvalu	0.0				1			1	1	1	
	Vanuatu	0.2				1					1	1
	Viet Nam	87.1	1	1	1	1	1	1	1	1	1	1

[a] Canada, the United States (American Region), Australia and Japan (Western Pacific Region) were not targeted, while in the European Region, only Armenia, Azerbaijan, Georgia, Kyrgyzstan, Tajikistan, Turkey, Turkmenistan, and Uzbekistan were targeted

[b] In millions, in 2008 (WHO 2010a)

Table 2. Global use of major insecticide compounds for control of malaria and dengue as reported to WHO, averaged over the period 2000–2009, and expressed in tonnes of active ingredient per year. The WHO recommended classification of pesticides by hazard is indicated (WHO 2010b).

Class of insecticide	Compound	WHO Hazard class[a]	Malaria	Dengue
Organochlorines	DDT	II	3604	0
Organophosphates	Malathion	III	620	214
	Dichlorvos (DDVP)	Ib	229	25
	Temephos	III	38	110
	Fenitrothion	II	31	21
	Other		42	25
Carbamates	Bendiocarb	II	23	0
	Propoxur	II	4	1
Pyrethroids	Cypermethrin	II	63	113
	Alpha-cypermethrin	II	55	10
	Deltamethrin	II	32	4
	Lambda-cyhalothrin	II	24	1
	Permethrin	II	19	11
	Other		25	15

[a] Ib, highly hazardous; II, moderately hazardous; III, slightly hazardous

Table 3. Trend in the use of insecticides for vector control in the WHO Regions, as reported to WHO, arranged by class of insecticide, and expressed in tonnes of active ingredient per year.

WHO Region	Class of insecticide[a]	2000	2001	2002	2003	2004	2005	2006	2007	2008	2009
African	OC	349	398	539	446	475	970	705	1,079	2,063	1,127
	OP	31	59	36	24	1	19	14	1	1	1
	C	1	1	0	5	6	14	11	19	21	23
	PY	4	10	11	14	4	43	14	14	29	98
American	OC	3	0	0	0	0	0	0	0	0	0
	OP	373	496	550	175	477	638	201	867	340	333
	C	8	1	1	5	3	2	0	1	13	9
	PY	134	198	1353	142	90	101	76	169	231	201
Eastern Mediterranean	OP	102	63	45	27	24	26	54	64	19	22
	C	3	2	4	1	0	1	3	13	15	0
	PY	5	13	9	12	26	19	33	27	18	16
European	OP	8	7	6	8	4	0	2	1	0	0
	C	0	0	0	0	0	0	2	0	0	0
	PY	0	1	1	2	1	3	4	4	1	1
South-East Asian	OC	3,160	4,906	4,274	5,246	5,270	5,094	3,732	3,479	4,107	4,000
	OP	5,177	161	119	32	96	139	378	125	91	164
	C	1	2	2	2	2	3	1	0	0	0
	PY	27	37	49	53	57	65	50	18	55	87
Western Pacific	OC	0	2	1	0	1	0	0	0	0	0
	OP	571	85	531	409	383	338	329	238	85	65
	PY	75	37	39	44	36	34	55	90	68	65

[a] OC, organochlorines; OP, organophosphates; C, carbamates; PY, pyrethroids

Table 4. Trend in the global use of insecticides targeted at the four major vector-borne diseases as reported to WHO, arranged by class of insecticide, and expressed in tonnes of active ingredient per year.[a]

Disease	Class of insecticide[b]	2000	2001	2002	2003	2004	2005	2006	2007	2008	2009
Malaria	OC	2,274	4,081	4,091	4,151	4,131	4,542	4,231	3,862	5,670	4,529
	OP	5,790	409	757	475	639	630	683	887	153	214
	C	8	3	3	6	11	19	16	32	36	23
	PY	132	130	484	143	133	156	113	109	151	256
Dengue	OP	313	390	487	175	325	480	216	317	302	306
	C	0	0	0	0	0	0	0	0	10	1
	PY	83	94	567	52	41	37	40	40	80	80
Leishma-niasis	OC	1,238	1,223	722	1,542	1,614	1,523	205	696	500	597
	OP	0	0	0	5	1	8	0	0	0	0
	C	3	2	3	1	0	0	0	0	0	2
	PY	7	13	129	9	8	4	7	8	23	15
Chagas disease	OP	8	4	4	0	4	15	0	0	0	0
	C	0	0	0	0	0	0	0	0	3	6
	PY	21	39	260	31	13	10	8	9	4	5

[a] Some countries reported that insecticide application methods were targeted at more than one disease. This mixed-purpose use of insecticides, which constituted 2% of the global use of organophosphates and 3% of global use of pyrethroids, was included in the assessment of insecticide use per disease.
[b] OC, organochlorines; OP, organophosphates; C, carbamates; PY, pyrethroids

References

WHO (World Health Organization). 2010a. World Health Statistics 2010. Geneva: World Health Organization. Available: http://www.who.int/whosis/whostat/2010/en/index.html [Accessed 10 January 2012]

WHO. 2010b. The WHO Recommended Classification of Pesticides by Hazard and Guidelines to Classification 2009. Geneva: World Health Organization. Available: http://www.who.int/ipcs/publications/pesticides_hazard/en/ [Accessed 10 January 2012]
